# Characterization of a *Cis*-Prenyltransferase from *Lilium longiflorum* Anther

**DOI:** 10.3390/molecules24152728

**Published:** 2019-07-26

**Authors:** Jyun-Yu Yao, Kuo-Hsun Teng, Ming-Che Liu, Co-Shine Wang, Po-Huang Liang

**Affiliations:** 1 Institute of Biological Chemistry, Academia Sinica, Taipei 115, Taiwan; 2 Institute of Biochemical Sciences, National Taiwan University, Taipei 106, Taiwan; 3 Graduate Institute of Biotechnology, National Chung Hsing University, Taichung 402, Taiwan

**Keywords:** isoprenoid, prenyltransferase, polyisoprenoid, dolichol, glycoprotein

## Abstract

A group of prenyltransferases catalyze chain elongation of farnesyl diphosphate (FPP) to designated lengths via consecutive condensation reactions with specific numbers of isopentenyl diphosphate (IPP). *cis*-Prenyltransferases, which catalyze *cis*-double bond formation during IPP condensation, usually synthesize long-chain products as lipid carriers to mediate peptidoglycan biosynthesis in prokaryotes and protein glycosylation in eukaryotes. Unlike only one or two *cis*-prenyltransferases in bacteria, yeast, and animals, plants have several *cis*-prenyltransferases and their functions are less understood. As reported here, a *cis*-prenyltransferase from *Lilium longiflorum* anther, named LLA66, was expressed in *Saccharomyces cerevisiae* and characterized to produce C40/C45 products without the capability to restore the growth defect from Rer2-deletion, although it was phylogenetically categorized as a long-chain enzyme. Our studies suggest that evolutional mutations may occur in the plant *cis*-prenyltransferase to convert it into a shorter-chain enzyme.

## 1. Introduction

Isoprenoids are natural products with different structures composed of C5 isopentenyl pyrophosphate (IPP) [1,2]. More than 80,000 naturally occurring isoprenoid molecules have been discovered, with a large number of new structures being reported each year [3]. IPP is synthesized through the classic mevalonate pathway from three molecules of acetyl coenzyme A, as well as in some bacteria and plants from a non-classic methylerythritol phosphate pathway [4,5]. IPP is then converted to its isomer dimethylallyl diphosphate (DMAPP) by IPP:DMAPP isomerase [6]. By condensation of DMAPP with one to three molecules of IPP, C10 geranyl diphosphate (GPP), C15 farnesyl diphosphate (FPP), and C20 genanylgeranyl diphosphate (GGPP) are synthesized by their respective synthases—GPPS, FPPS, and GGPPS. These short chain-length products serve as precursors, leading to a variety of natural isoprenoids, such as sterols, carotenoids, dolichols, ubiquinones, and prenylated proteins, functioning as hormones, visual pigments, constituents of membranes, and components of signal transduction [7].

**Figure 1:**
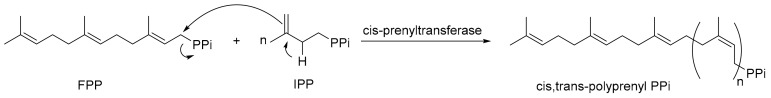
The reactions catalyzed by cis-prenyltransferases.

A group of prenyltransferases catalyze condensation of specific numbers of IPP with FPP to generate linear polymers with defined chain lengths [8]. These prenyltransferases are classified as *cis*- or *trans*-type, according to the stereochemistry of double bonds resulting from IPP condensation. *Trans*-prenyltransferases synthesize products up to C50, which serve as ligands for posttranslational modifications of signaling proteins, ether-linked lipids in thermophilic *archaea*, and side chains of ubiquinones [7]. In contrast, *cis*-prenyltransferases usually catalyze products >C50 (see Figure 1 for the reactions). Among these *cis*-prenyltransferases, bacterial undecaprenyl diphosphate synthase (UPPS) generates C55 product to serve as a lipid carrier for cell wall peptidoglycan biosynthesis [9]. Its homologous *cis*-prenyltransferases in eukaryotes such as yeast Rer2 and Srt1 [10] and human dehydrodolichyl diphosphate synthase (DHDDS) [11] synthesize C70–120 polyisoprenoids that are subsequently converted by reductases to dolichols for glycoprotein biosynthesis [12]. While bacteria, yeast, and humans have only one or two *cis*-prenyltransferases, plants have several—for example, *Arabidopsis thaliana* contains nine cis-prenyltransferases (*At*CPTs), and six out of nine putative genes are expressed in roots [13]; tomatoes have seven *Sl*CPTs [14]. The biological functions of polyisoprenoids in plants are largely unknown, although some functions have been postulated [15]. 

A *cis*-prenyltransferase, LLA66, was identified from a subtractive cDNA library during microspore development in the *L. longiflorum* anther [16]. The predicted amino acid sequence of LLA66 shares 30–41% of its identity with *cis*-prenyltransferases from various species (Supplementary Figure S1). The prenyltransferase activity in the microspores correlates with tapetal development; however, no in vitro biochemical characterization has been performed. As reported herein, we have expressed LLA66 in *S. cerevisiae* and characterized its products in *rer2*-deleted (*rer2*Δ) yeast and from the purified recombinant protein. Moreover, a yeast protein complex was co-purified to show an IPP-utilizing activity to convert IPP into farnesol. 

## 2. Results and Discussion

### 2.1. Products and Functional Analysis of LLA66 in S. cerevisiae

**Figure 2:**
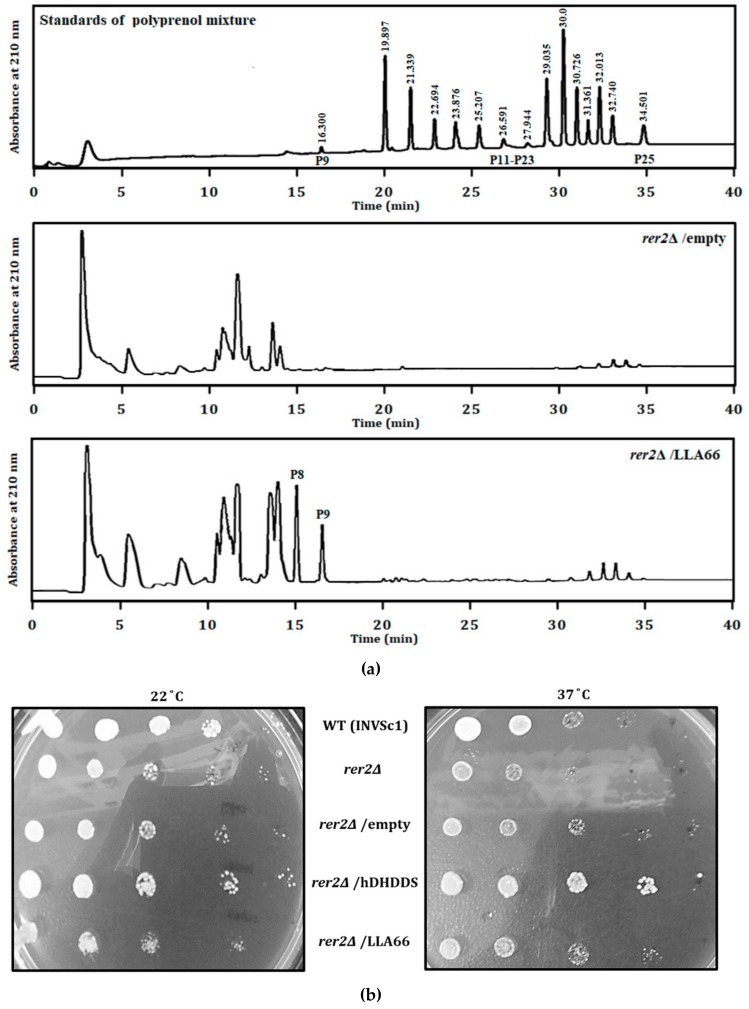
Analysis of LLA66 products and their ability in rescuing the growth of *rer2*Δ mutant yeast cells. (**a**) Standards (top) and polyisoprenoids isolated from the *rer2*Δ mutant yeast transformed with the pYES2 vector (middle) or the pYES2-LLA66 plasmid (bottom). The extra peaks at the elution time of 15 and 16.6 min produced by LLA66 were P8 and P9, as compared to the standards. (**b**) The colonies of *S. cerevisiae* wild type (WT), *rer2*Δ mutant, and the *rer2*Δ mutant transformed with the vector pYES2 (empty), pYES2-DHDDS, and pYES2-LLA66, respectively, were examined. Five-fold serially diluted yeast cultures starting from OD_600_ of 0.4 were plated on solid YP-2% galactose medium and incubated at 22 °C (left) or 37 °C (right) for five days.

To determine the function of LLA66, it was expressed in a *rer2*Δ mutant yeast lacking the Rer2 *cis*-prenyltransferase activity. Comparing that to the normal *rer2*Δ yeast, *rer2*Δ yeast expressing LLA66 synthesized C40 and C45 extra products composed of 8 and 9 isoprene units, respectively (Figure 2a). The *rer2*Δ yeast showed temperature-sensitive growth. Transformation with pYES2-LLA66, similar to the empty vector, could not restore growth of the *rer2*Δ mutant (Figure 2b). In contrast, the human homologue of yeast Rer2, DHDDS restored the growth of the *rer2*Δ mutant (Figure 2b). The above observations suggested that LLA66 was not capable of supporting glycoprotein biosynthesis because it synthesized shorter-chain products.

### 2.2. Products of the Recombinant LLA66

**Figure 3:**
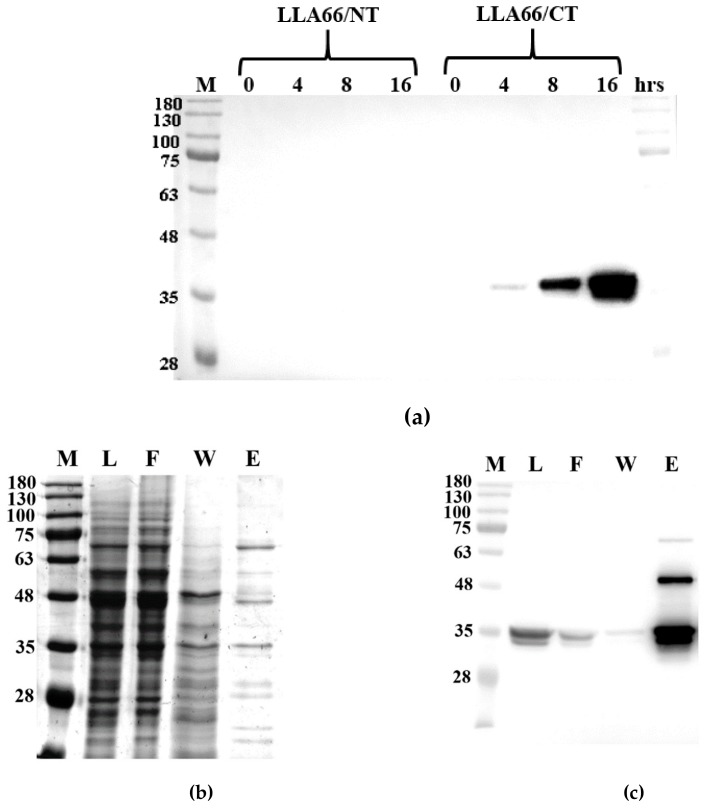
Purification of the recombinant LLA66. (**a**) Anti-His-tag western blot analysis of LLA66 linked with N- or C-terminal His-tag after different induction time periods. (**b**) SDS-PAGE analysis of the Ni-NTA purified LLA66. (**c**) Anti-His-tag western blot analysis of the Ni-NTA purified LLA66. M = markers, L = lysis supernatant, F = flow through, W = washed with 20 mM imidazole, and E = eluted with 300 mM imidazole.

LLA66 was first expressed with *E. coli*, but failed to yield a soluble protein. Then, the enzyme was overexpressed in a *S. cerevisiae* INVSc1 strain, which is a fast-growing diploid strain ideal for expression by using a pYES2 vector that encodes a His-tag. The expression vector has the yeast *GAL1* promoter for a high level of protein induction by galactose and repression by glucose, as well as the *URA3* gene for selection of transformants in yeast host strains with a *ura3* genotype. The His-tag was attached to the N or C terminus, but only C-terminally His-tagged LLA66 was soluble (Figure 3a). After the yeast was induced with 2% galactose, the cells were harvested and disrupted by a French press instrument. Immunoblotting against the recombinant LLA66 with a His-tag antibody revealed a molecular mass of 35.5 kDa band in the supernatant and in the Ni-NTA agarose-purified fractions (Figure 3b,c).

**Figure 4:**
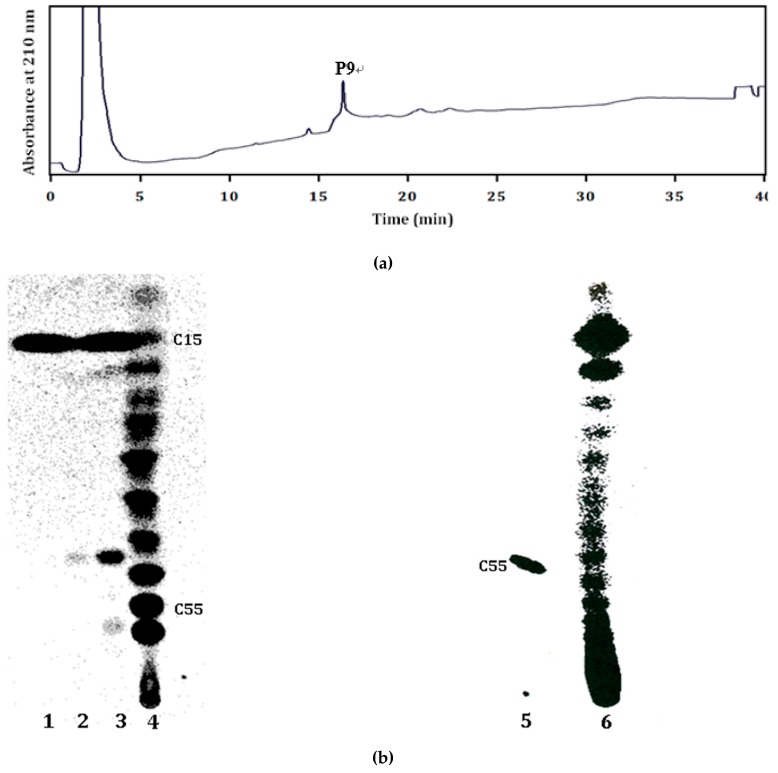
HPLC and TLC analysis of polyisoprenoids from recombinant LLA66 after Ni-NTA purification. (**a**) The C45 product extracted from the mixture of purified LLA66 with substrates FPP and IPP was eluted at 15.9 min under HPLC analysis. (**b**) C15 product was produced by using [^14^C]IPP and FPP with *S. cerevisiae* cell lysate after Ni-NTA purification, as shown in lane 1. C15 and/or C45 products were generated by using [^14^C]IPP alone (lane 2) or [^14^C]IPP and FPP (lane 3) with *S. cerevisiae* lysate expressing LLA66 after Ni-NTA purification. C20–60 products were produced by using [^14^C]IPP and FPP with the purified *E. coli* UPPS in the absence of Triton X-100, as shown in lane 4. C55 was produced by using [^14^C]IPP and FPP with *E. coli* UPPS in the presence of 0.1% Triton X-100, as shown in lane 5. Products with a variety of different chain lengths were generated by human DHDDS, as shown in lane 6.

Products of the purified recombinant LLA66 with FPP and IPP substrates were analyzed by HPLC. As shown in Figure 4a, the peak that eluted at 15.9 min corresponded to a C45 product under the same HPLC condition for analyzing the in vivo products of LLA66 in yeast. When the products where FPP and [^14^C]IPP were used as substrates were examined by TLC, a band corresponding to the C45 chain length was found (Figure 4b, lane 3), as compared to the C20–60 products generated by *E. coli* UPPS (lane 4) expressed in *E. coli* [17], consistent with the HPLC analysis. However, an extra band for C15 product was also detected (lane 3), even when only [^14^C]IPP was used as the substrate (lane 2). To investigate whether this C15 FPP was formed by LLA66 or yeast enzymes, we grew the yeast without LLA66 plasmid (pYES2-empty) and performed the same Ni-NTA chromatography. We found the fractions eluted by 300 mM imidazole contained the IPP-utilizing activity, and the product was C15 (lane 1). Therefore, this activity was due to an enzyme mixture binding to Ni-NTA. We further purified such an enzyme mixture through other columns (see below). To confirm that *S. cerevisiae* is a suitable host to express long-chain *cis*-prenyltransferases, we also expressed human DHDDS and showed that the recombinant protein was active and produced expected long-chain products (lane 6) as compared to the C55 UPPS product generated in the presence of 0.1% Triton X-100 (lane 5). 

### 2.3. Identification of S. cerevisiae Enzymes for Unexpected IPP-Utilizing Activity

We cultured INVSc1 yeast without LLA66 and performed DEAE ion exchange chromatography to partially purify the enzyme mixture. The activity of using [^14^C]IPP alone was detected at 200 to 300 mM NaCl eluting fractions (Supplementary Figure S2a). For the next chromatographic step, the yeast enzyme mixture with the activity was bound to Ni-NTA and eluted by at least 100 mM imidazole (Supplementary Figure S2b). Then, a strong anion exchange chromatography (Supplementary Figure S2c), followed by a size exclusion chromatography (Supplementary Figure S2d) were used to reveal an active protein peak. SDS-PAGE of the fractions after three purification steps is shown in Supplementary Figure S3a. Finally, after size exclusion chromatography, bands of approximately 60, 40, and 30 kDa of the active protein peak resolved on SDS-PAGE (Supplementary Figure S3b) were subjected to LC-MASS/MASS analysis. These proteins were identified to be alkaline phosphatase, FPPS and IPP:DMAPP isomerase (Supplementary Figure S4 shows the detected fragmented peptides in red from the protein bands, and Table S1 lists the identified proteins). This could explain that adding [^14^C]IPP alone generated C15 product, as shown by TLC analysis, because IPP was converted to DMAPP by the isomerase, which was condensed with two IPP molecules to generate FPP by FPPS, and finally, the phosphatase hydrolyzed FPP to C15 farnesol. This protein complex could strongly bind to NiNTA, probably due to the high content of His (26 His) in alkaline phosphatase. This is worth noticing because others could experience the same problem when expressing prenyltransferases using *S. cerevisiae*. 

### 2.4. Phylogenetic Tree and Homology-Based Model of LLA66

**Figure 5:**
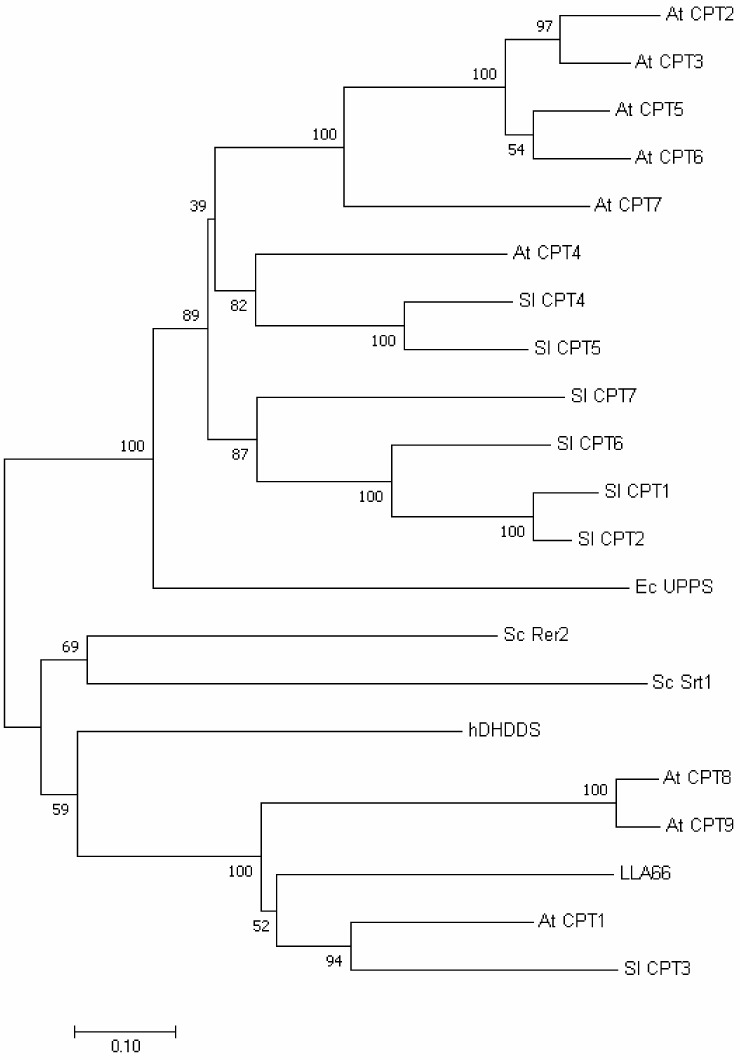
Phylogenetic tree analysis. Data showing phylogenetic distances of LLA66 from other *cis*-prenyltransferases in human, *A. thaliana*, *Solanum lycopersicum*, *S. cerevisiae*, and *E. coli.*

**Figure 6:**
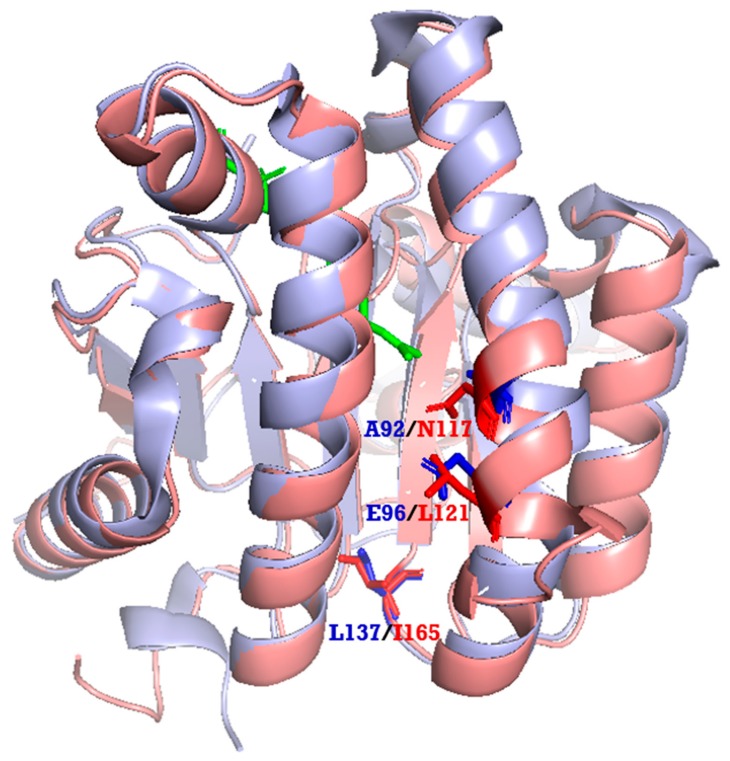
Superimposition of the homology-based model of LLA66 and *E. coli* UPPS structure. The magenta ribbon is the model of LLA66 based on the *S. aureus* UPPS structure (PDB code: 4H8E). The light blue ribbon is the *E. coli* UPPS structure (PBD code: 1V7U). N117 and/or L121 in the LLA66 model could limit the product chain length, because the corresponding residues are smaller A92 and E96 in *E. coli* UPPS, respectively. A large amino acid L137 located at the bottom of *E. coli* UPPS was proposed to limit the C55 product [17]. Green stick represents the substrate FPP.

We also performed phylogenetic tree analysis and found that LLA66 was more homologous to long-chain *cis*-prenyltransferases (Figure 5), unlike the C40/C45 products identified in LLA66-expressing yeast. To explain this discrepancy, we generated the homology-based model of LLA66 (Figure 6) based on the structure of *S. aureus* UPPS. When the LLA66 model is superimposed and compared with the structure of *E. coli* UPPS, we found that the large amino acids, such as N117 and/or L121 in the LLA66 structure, could limit the final chain lengths. The corresponding residues in *E. coli* UPPS structure are smaller Ala92 and Glu96, respectively, in the middle of the active site, while L137 at the bottom was shown to determine the C55 product [17]. Therefore, LLA66 could be evolved from a long-chain *cis*-prenyltransferase in this plant through the putative mutation(s). 

In eukaryotes, dolichols of C70–120 play indispensable roles as glycosyl carrier lipids for the biosynthesis of glycoproteins in the endoplasmic reticulum. For this function, one human *cis*-prenyltransferase (DHDDS) and two yeast *cis*-prenyltransferases (Rer2 and Srt1), which can synthesize long-chain products, are known. Rer2 was found to be the major player for glycoprotein biosynthesis in yeast. We demonstrate here that LLA66 produced C40 and C45 products in *S. cerevisiae*, but could not rescue the growth defect of the *rer2*Δ mutant because it failed to make a long enough lipid for protein glycosylation in yeast. Because the genome of *L. longiflorum* is not sequenced, LLA66 is the only identified *cis*-prenyltransferase so far. Judging from long-chain glycosyl carrier lipids and several *cis*-prenyltransferases in other plants, there are likely other *cis*-prenyltransferases, but not LLA66, responsible for biosynthesis of glycoproteins in *L. longiflorum*. 

In addition to dolichols, seed plants produce other types of *Z*,*E*-mixed polyisoprenoids, such as ficaprenol (tri-*trans*, poly-*cis*-polyprenol, C45–75) and betulaprenol (di-*trans*, poly-*cis*-polyprenol, C30–45 and ≥C70) in abundance [15]. For the nine *cis*-prenyltransferases identified from the genome of *A. thaliana*, one expressed in *E. coli* produced a C130 long-chain product [18]. Others yield shorter products, including a C80 by LEW1 expressed in *E. coli* [19], a C35 *cis*, *trans*-mixed heptaprenyl diphosphate by the synthase expressed in *E. coli* [20], and a mixture of polyprenols with a dominating 7-isoprene prenol (C35) by *At*CPT6 expressed in *S. cerevisiae* [21]. 

Similarly, various products were ascribed to the seven tomato *Sl*CPTs [a single prenol in a range Pren-2 (C10) to Pren-4 (C20) for *Sl*CPT1, *Sl*CPT2, and *Sl*CPT6, a single Pren-13 (C65) for *Sl*CPT3, and medium-chain-length products, Pren-5 (C25) to Pren-13 (C65) with no clear distribution profile for *Sl*CPT4, *Sl*CPT5, and *Sl*CPT7] [14]. Interestingly, since only one of the tomato CPTs, *Sl*CPT3, was shown to complement the growth defect of the *rer2*Δ mutant, only this enzyme has been postulated to be involved in dolichol biosynthesis. RNAi-mediated suppression of *Sl*CPT3 resulted in a ~60% decrease in dolichol content. However, the involvement of *Sl*CPT3 in dolichol biosynthesis requires the participation of a distantly related partner protein, designated as a CPT-binding protein (*Sl*CPTBP), which is a close homolog of the human Nogo-B receptor for supporting DHDDS activity [22]. A very short-chain C10 neryl diphosphate, produced from condensation of IPP with DMAPP catalyzed by a *cis*-prenyltransferase, has been identified in tomatoes, serving as a precursor to monoterpenes [23]. 

LLA66 is regulated by gibberellin during the development of lily anthers [24], but the real function is still unknown. It is phylogenetically distinct from other monocot *cis*-prenyltransferases [16], but more homologous to long-chain *cis*-prenyltransferases, such as *Sl*CPT3, that is responsible for dolichol synthesis, as shown in our phylogenetic tree analysis. From the structural model, the shorter product may come from the large amino acids in the middle of the active site. We suspect this enzyme is an evolutional mutant from a long-chain *cis*-prenyltransferase in the plant. More in vivo and in vitro characterizations of plant *cis*-prenyltransferases should increase our understanding on their biological functions, as well as explain why plants require more *cis*-prenyltransferases than bacteria, yeast, and animals.

## 3. Materials and Methods

### 3.1. Materials

IPP, FPP, potato acid phosphatase, and Ni-NTA resin were obtained from Sigma (St. Louis, MO, USA). Reverse-phase TLC was purchased from Merck (Kenilworth, NJ, USA). PrimeSTAR^®^ GXL DNA polymerase for PCR was obtained from Takara (Shiga, Japan). The plasmid mini-prep kit and DNA gel extraction kit were purchased from Qiagen (Hilden, Germany). All commercial buffers and reagents were of the highest grade. 

### 3.2. Preparation of LLA66 Construct

The LLA66-encoding gene was synthesized by Invitrogen with codons optimized for *S. cerevisiae* expression. The forward primer (5′- GGGAATATTAAGCTTAACAAAATGATCTCCCACGAATTGTC-3′) with a HindIII site and the reverse primer (5′-AGATGCATGCTCGAGTTAGAATTGAATGTAG-3′) containing an XhoI site, were used to amplify the gene. PCR was performed in a final volume of 50 μL containing 20 pmol of an amplification primer pair for 30 cycles at 30 s at 98 °C, 60 s at 55 °C, and 1.5 min at 68 °C, with a 3 min preheat at 98 °C and a 10 min final extension at 68 °C. The PCR product was subjected to agarose gel electrophoresis in TAE buffer and stained with ethidium bromide. The part of the gel containing the band of the correct size—about 1 k-bp—was excised, and the DNA was recovered using a DNA extraction kit. The purified DNA was ligated into the HindIII/XhoI-digested pYES2/CT expression vector (Invitrogen) by incubation for 4 h at 22 °C to generate the expression plasmid pYES2/CT-LLA66. 

### 3.3. Extraction of Polyisoprenoids from S. cerevisiae

The correct pYES2-LLA66 construct or the vector-only pYES2 was transformed to INVSc1 or *rer2*Δ competent cells. The 10 mL overnight culture of a single transformant was used to inoculate 400 mL of fresh SC minimal medium without uracil. An appropriate amount of culture was centrifuged at 1500× *g* for 5 min and resuspended in fresh induction medium containing 2% galactose to give a final OD_600_ of 0.4. The medium was then grown at 30 °C for 24 h with shaking. Stationary-phase yeast cells grown at 30 °C were harvested by centrifugation at 1500× *g* for 5 min at 4 °C and washed once with sterile water. The pellets were dissolved in 10 mL of hydrolytic solution (25 g of KOH was dissolved in 35 mL of sterile distilled water, and brought to 100 mL with 99.8% ethanol), vortexed for 1 min, and incubated at 95 °C for 1 h. Non-saponifiable lipids were then extracted three times with hexane. The pooled extracts were evaporated and purified on a silica gel 60 column using an isocratic elution with 10% diethyl ether in hexane. Purified polyisoprenoids were analyzed by HPLC/UV using external and internal standards of different chain lengths for comparison by following the procedure [21].

### 3.4. HPCL Analysis of the LLA66 Products in S. cerevisiae

For HPLC analysis, an established protocol was used [21]. A dual-pump HPLC device (Hewlett-Packard) was used with a ZORBAX XDB-C18 (4.6 × 75 mm, 3.5 μm) reversed-phase column (Agilent, Santa Clara, CA, USA) eluted by a solvent ingredient from 100% buffer A (methanol/water, 9:1 by vol.) to 100% buffer B (methanol/propane-2-ol/hexane, 2:1:1 by vol.) in 40 min at a flow rate of 1.5 mL/min. The chain lengths of lipids were confirmed by comparing with a polyprenol mixture of Pren-9, 11–23, and 25 as external standards. 

### 3.5. Expression and Purification of the Recombinant LLA66

The correct construct was transformed to INVSc1 competent cells (Invitrogen, Waltham, MA, USA) for protein expression by *S. cerevisiae* using an EasyComp Transformation Kit (Invitrogen). The 10 mL overnight culture of a single transformant was used to inoculate 400 mL of fresh SC minimal medium without uracil. An appropriate amount of culture was centrifuged at 1500× *g* for 5 min and resuspended in fresh induction medium containing 2% galactose to give a final OD_600_ of 0.4. The medium was then grown at 30 °C for 24 h with shaking. The cells were washed with sterile water and harvested by centrifugation at 1500× *g* for 5 min. Cell pellets were suspended in 75 mL of lysis buffer [25 mM Tris-HCl (pH 7.5) and 150 mM NaCl]. A French press instrument (Constant Cell Disruption System) was used to disrupt the cells at 25,000 p.s.i., followed by centrifugation at 100,000× *g* for 1 h. The supernatant was loaded onto a 10 mL NiNTA column, which was equilibrated with the lysis buffer. The column was washed with the lysis buffer containing 50 mM imidazole and eluted with the buffer containing 300 mM imidazole. Fractions collected were analyzed by SDS-PAGE and western blot. Protein purification was performed at 4 °C.

### 3.6. TLC Analysis of the Recombinant LLA66 Products

For product analysis, the enzymatic reactions containing LLA66 enzyme, [^14^C]IPP, and FPP in the reaction buffer (0.5 mM MgCl_2_, 50 mM KCl, and 100 mM Hepes, pH 7.5, with or without 0.1% Triton X-100) were performed for 24 h at 25 °C. The radiolabeled polyprenyl pyrophosphates were extracted with 1-butanol. After removal of 1-butanol by evaporation, polyprenyl pyrophosphates were converted to polyprenols by using a commercial acidic pyrophosphatase. The radiolabeled polyprenols were extracted with n-hexane, and the volume was reduced by evaporation. The n-hexane solution of radiolabeled polyprenols were spotted on a reversed-phase TLC plate, and the plate was then developed using a mixture of acetone and water at a 19:1 ratio by volume as the mobile phase. The plate with radiolabeled products was analyzed by autoradiopraphy using a bioimaging analyzer (Bio-Rad).

### 3.7. Phylogenetic Tree Analysis of LLA66

BioEdit and Mega6 were used for phylogenetic analysis of LLA66 and other *cis*-prenyltransferases from human, *A. thaliana*, *Solanum lycopersicum*, *S. cerevisiae*, and *E. coli* via the neighbor-joining algorithm. 

### 3.8. Homology Modeling of LLA66

The SWISS-MODEL server indicated that the closest homology to LLA66 was UPPS of *S. aureus* (PDB code: 4H8E). Therefore, 4H8E was used as a template to generate a structural model for a fragment (L37–A277) of LLA66.

## Supplementary Materials

The followings are available on-line, Figure S1: Sequence homology of LLA66 with other *cis*-prenyltransferases, including UPPS from *E. coli*, Rer2 and Srt1 from yeast, DHDDS from human, and 9 *cis*-prenyltransferases from *A. thaliana*; Figure S2: Purification of the *S. cerevisiae* enzyme complex that produced C15 farnesol from [^14^C]IPP alone; Figure S3: SDS-PAGE analysis of the *S. cerevisiae* enzyme complex from a series of purification steps; Figure S4: Identification of the proteins from LC-MS/MS analysis; Table S1: Proteins identified by LC-MS/MS analysis from the protease digested SDS-PAGE bands.

## Abbreviations

IPP	isopentenyl diphosphate
DMAPP	dimethylallyl diphosphate
FPP	farnesyl diphosphate
FPPS	farnesyl diphosphate synthase
UPPS	undecaprenyl diphosphate synthase
PCR	polymerase chain reaction
Ni-NAT	nickel nitrilo-tri-acetic acid
SDS-PAGE	sodium dodecyl sulfate-polyacrylamide
Da.	Dalton
IPTG	isopropyl-β-d-thiogalactopytanoside
Tris	tris(hydroxymethyl)aminomethane
Hepes	4-(2-hydroxyethyl)-1-piperazineethanesufonic acid
HPLC	high performance liquid chromatography
TLC	thin layer chromatography

## References

[1] Poulter C.D., Rilling H.C., Porter J.W., Spurgeon S.L. (1981). Biosynthesis of Isoprenoid Compounds.

[2] Ogura K., Koyama T., Sagami H., Bittman R. (1997). Subcellular Biochemistry.

[3] Sacchettini J.C., Poulter C.D. (1997). Creating isoprenoid diversity. Science.

[4] Rohmer M., Knani M., Simonin P., Sutter B., Sahm H. (1993). Isoprenoid biosynthesis in bacteria: A novel pathway for the early steps leading to isopentenyl diphosphate. Biochem. J..

[5] Arigoni D., Sagner S., Latzel C., Eisenreich W., Bacher A., Zenk M.H. (1997). Terpenoid biosynthesis from 1-deoxy-d-xylulose in higher plants by intramolecular skeletal rearrangement. Proc. Natl. Acad. Sci. USA.

[6] Durbecq V., Sainz G., Oudjama Y., Clantin B., Bompard-Gilles C., Tricot C., Caillet J., Stalon V., Droogmans L., Villeret V. (2001). Crystal structure of isopentenyl diphosphate:dimethylallyl diphosphate isomerase. EMBO J..

[7] Liang P.H. (2009). Reaction kinetics, catalytic mechanisms, conformational changes, and inhibitor design for prenyltransferases. Biochemistry.

[8] Wang K., Ohnuma S. (1999). Chain-length determination mechanism of isoprenyl diphosphate synthases and implications for molecular evolution. Trend Biochem. Sci..

[9] Teng K.H., Liang P.H. (2012). Undecaprenyl diphosphate synthase, a *cis*-prenyltransferase synthesizing lipid carrier for bacterial cell wall biosynthesis. Mol. Membr. Biol..

[10] Sato M., Sato K., Nishikawa S., Hirata A., Kato J., Nakano A. (1999). The yeast RER2 gene, identified by endoplasmic reticulum protein localization mutations, encodes *cis*-prenyltransferase, a key enzyme in dolichol synthesis. Mol. Cell. Biol..

[11] Endo S., Zhang Y.W., Takahashi S., Koyama T. (2003). Identification of human dehydrodolichyl diphosphate synthase gene. Biochim. Biophys. Acta.

[12] Jozwiak A., Gutkowska M., Gawarecka K., Surmacz L., Buczkowska A., Lichocka M., Nowakowska J., Swiezewska E. (2015). Polyprenol Reductase2 deficiency is lethal in Arabidopsis due to male sterility. Plant Cell.

[13] Surmacz L., Swiezewska E. (2011). Polyisoprenoids: Secondary metabolites or physiologically important superlipids?. Biochem. Biophys. Res. Commun..

[14] Akhtar T.A., Matsuba Y., Schauvinhold I., Yu G., Lees H.A., Klein S.E., Pichersky E. (2013). The tomato *cis*-prenyltransferase gene family. Plant J..

[15] Swiezewska E., Danikiewicz W. (2005). Polyisoprenoids: Structure, biosynthesis and function. Prog. Lipid Res..

[16] Liu M.C., Wang B.J., Huang J.K., Wang C.S. (2011). Expression, localization and function of a *cis*-prenyltransferase in the Tapetum and Microspores of Lily Anthers. Plant Cell Physiol..

[17] Ko T.P., Chen Y.K., Robinson H., Tsai P.C., Gao Y.G., Chen A.P., Wang A.H., Liang P.H. (2001). Mechanism of Product Chain Length Determination and the Role of a Flexible Loop in *Escherichia coli* Undecaprenyl-pyrophosphate Synthase Catalysis. J. Biol. Chem..

[18] Oh S.K., Han K.H., Ryu S.B., Kang H. (2000). Molecular cloning, expression, and functional analysis of a *cis*-prenyltransferase from *Arabidopsis thaliana*. Implications in rubber biosynthesis. J. Biol. Chem..

[19] Zhang H., Ohyama K., Boudet J., Chen Z., Yang J., Zhang M., Muranaka T., Maurel C., Zhu J.K., Gong Z. (2008). Dolichol biosynthesis and its effects on the unfolded protein response and abiotic stress resistance in Arabidopsis. Plant Cell.

[20] Kera K., Takahashi S., Sutoh T., Koyama T., Nakayama T. (2012). Identification and characterization of a *cis*, *trans*-mixed heptaprenyl diphosphate synthase from *Arabidopsis thaliana*. FEBS J..

[21] Surmacz L., Plochocka D., Kania M., Danikiewicz W., Swiezewska E. (2014). *cis*-Prenyltransferase atCPT6 produces a family of very short-chain polyisoprenoids in planta. Biochim. Biophys. Acta.

[22] Brasher M.I., Surmacz L., Leong B., Pitcher J., Swiezewska E., Pichersky E., Akhtar T.A. (2015). A two-component enzyme complex is required for dolichol biosynthesis in tomato. Plant J..

[23] Schilmiller A.L., Schauvinhold I., Larson M., Xu R., Charbonneau A.L., Schmidt A., Wilkerson C., Last R.L., Pichersky E. (2009). Monoterpenes in the glandular trichomes of tomato are synthesized from a neryl diphosphate precursor rather than geranyl diphosphate. Proc. Natl. Acad. Sci. USA.

[24] Hsu Y.F., Tzeng J.D., Liu M.C., Yei F.L., Chung M.C., Wang C.S. (2008). Identification of anther-specific/predominant genes regulated by gibberellin during development of lily anthers. J. Plant Physiol..

