# Supplementary material for: Characterization of a Cis-Prenyltransferase from Lilium longiflorum Anther
Source: Molecules. 2019 Jul 26;24(15):2728. doi: 10.3390/molecules24152728 (PMC6696123; doi:10.3390/molecules24152728)
Supplement: Supplementary file 1 [file molecules-24-02728-s001.pdf]

molecules

MDPI

Supplementary Materials

Characterization of a *Cis*-Prenyltransferase from *Lilium Longiflorum* Anther

Jyun-Yu Yao [1,2], Kuo-Hsun Teng [1,2], Ming-Che Liu [3], Co-Shine Wang [3] and Po-Huang Liang [1,2,*]

[1] Institute of Biological Chemistry, Academia Sinica, Taipei 115, Taiwan
[2] Institute of Biochemical Sciences, National Taiwan University, Taipei 106, Taiwan
[3] Graduate Institute of Biotechnology, National Chung Hsing University, Taichung 402, Taiwan
* Correspondence: phliang@gate.sinica.edu.tw; Tel.: 886-2-3366-4069; Fax: 886-2-2363-5038

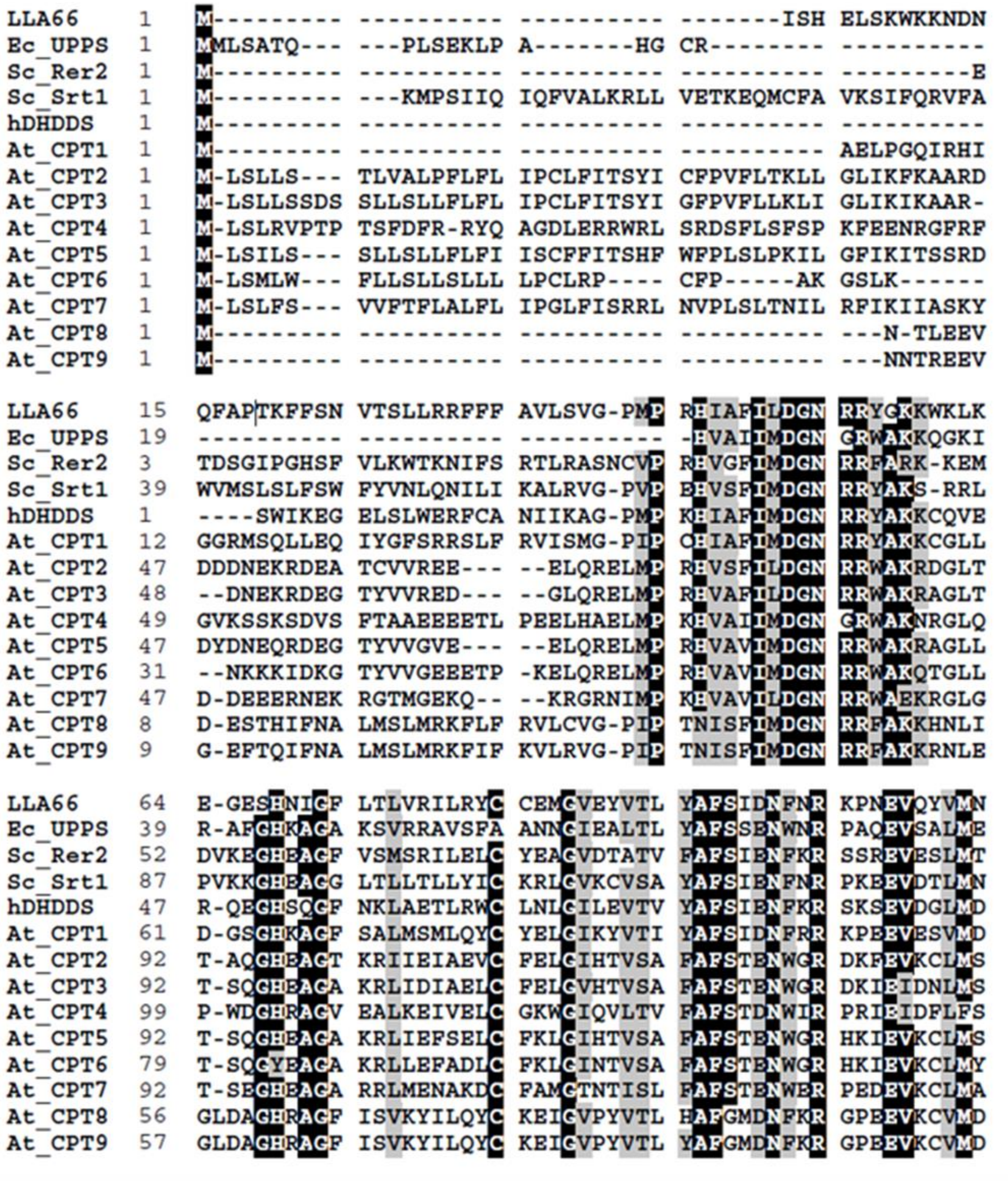

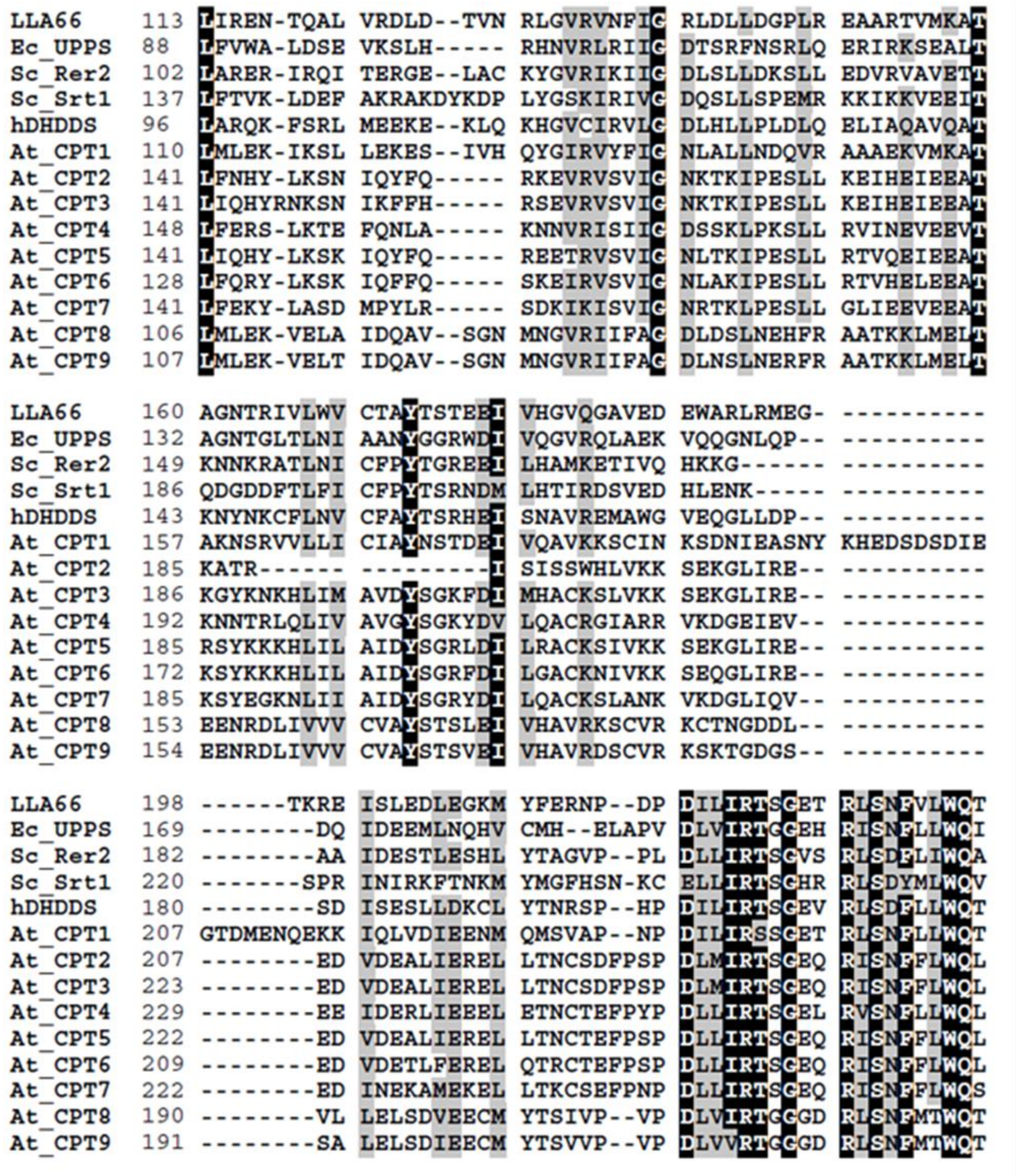
LLA66 113 LIREN-TQAL VRDLD--TVN RLGVRVNFIG RLDLLDGPLR EAARTVMKAT
Ec_UPPS 88 LFVWA-LDSE VKSLH----- RHNVRLRIIG DTSRFNSRLQ ERIRKSEALT
Sc_Rer2 102 LARER-IRQI TERGE--LAC KYGVRIKIIG DLSLLDKSLL EDVRVAVETT
Sc_Srt1 137 LFTVK-LDEF AKRAKDYKDP LYGSKIRIVG DQSLLSPEMR KKIKKVEEIT
hDHDDS 96 LARQK-FSRL MEEKE--KLQ KHGVCIRVLG DLHLLPLDLQ ELIAQAVQAT
At_CPT1 110 LMLEK-IKSL LEKES--IVH QYGIRVYFIG NLALLNDQVR AAAEKVMKAT
At_CPT2 141 LFNHY-LKSN IQYFQ----- RKEVRVSVIG NKTKIPESLL KEIHEIEEAT
At_CPT3 141 LIQHYRNKSN IKFFH----- RSEVRVSVIG NKTKIPESLL KEIHEIEEAT
At_CPT4 148 LFERS-LKTE FQNLA----- KNNVRISIIG DSSKLPKSLL RVINEVEEVT
At_CPT5 141 LIQHY-LKSK IQYFQ----- REETRVSVIG NLTKIPESLL RTVQEIEEAT
At_CPT6 128 LFQRY-LKSK IQFFQ----- SKEIRVSVIG NLAKIPESLL RTVHELEEAT
At_CPT7 141 LFEKY-LASD MPYLR----- SDKIKISVIG NRTKLPESLL GLIEEVEEAT
At_CPT8 106 LMLEK-VELA IDQAV--SGN MNGVRIIFAG DLDSLNEHFR AATKKLMELT
At_CPT9 107 LMLEK-VELT IDQAV--SGN MNGVRIIFAG DLNSLNERFR AATKKLMELT
LLA66 160 AGNTRIVLWV CTAYTSTEEI VHGVQGAVED EWARLRMEG- ----------
Ec_UPPS 132 AGNTGLTLNI AANYGGRWDI VQGVRQLAEK VQQGNLQP-- ----------
Sc_Rer2 149 KNNKRATLNI CFPYTGREEI LHAMKETIVQ HKKG------ ----------
Sc_Srt1 186 QDGDDFTLFI CFPYTSRNDM LHTIRDSVED HLENK----- ----------
hDHDDS 143 KNYNKCFLNV CFAYTSRHEI SNAVREMAWG VEQGLLDP-- ----------
At_CPT1 157 AKNSRVVLLI CIAYNSTDEI VQAVKKSCIN KSDNIEASNY KHEDSDSDIE
At_CPT2 185 KATR------ ---------I SISSWHLVKK SEKGLIRE-- ----------
At_CPT3 186 KGYKNKHLIM AVDYSGKFDI MHACKSLVKK SEKGLIRE-- ----------
At_CPT4 192 KNNTRLQLIV AVGYSGKYDV LQACRGIARR VKDGEIEV-- ----------
At_CPT5 185 RSYKKKHLIL AIDYSGRLDI LRACKSIVKK SEKGLIRE-- ----------
At_CPT6 172 KSYKKKHLIL AIDYSGRFDI LGACKNIVKK SEQGLIRE-- ----------
At_CPT7 185 KSYEGKNLII AIDYSGRYDI LQACKSLANK VKDGLIQV-- ----------
At_CPT8 153 EENRDLIVVV CVAYSTSLEI VHAVRKSCVR KCTNGDDL-- ----------
At_CPT9 154 EENRDLIVVV CVAYSTSVEI VHAVRDSCVR KSKTGDGS-- ----------
LLA66 198 ------TKRE ISLEDLEGKM YFERNP--DP DILIRTSGET RLSNFVLWQT
Ec_UPPS 169 --------DQ IDEEMLNQHV CMH--ELAPV DLVIRTGGEH RISNFLLWQI
Sc_Rer2 182 --------AA IDESTLESHL YTAGVP--PL DLLIRTSGVS RLSDFLIWQA
Sc_Srt1 220 -------SPR INIRKFTNKM YMGFHSN-KC ELLIRTSGHR RLSDYMLWQV
hDHDDS 180 --------SD ISESLLDKCL YTNRSP--HP DILIRTSGEV RLSDFLLWQT
At_CPT1 207 GTDMENQEKK IQLVDIEENM QMSVAP--NP DILIRSSGET RLSNFLLWQT
At_CPT2 207 --------ED VDEALIEREL LTNCSDFPSP DLMIRTSGEQ RISNFFLWQL
At_CPT3 223 --------ED VDEALIEREL LTNCSDFPSP DLMIRTSGEQ RISNFFLWQL
At_CPT4 229 --------EE IDERLIEEEL ETNCTEFPYP DLLIRTSGEL RVSNFLLWQL
At_CPT5 222 --------ED VDEALIEREL LTNCTEFPSP DLLIRTSGEQ RISNFFLWQL
At_CPT6 209 --------ED VDETLFEREL QTRCTEFPSP DLLIRTSGEQ RISNFFLWQL
At_CPT7 222 --------ED INEKAMEKEL LTKCSEFPNP DLLIRTSGEQ RISNFFLWQS
At_CPT8 190 --------VL LELSDVEECM YTSIVP--VP DLVIRTGGGD RLSNFMTWQT
At_CPT9 191 --------SA LELSDIEECM YTSVVP--VP DLVVRTGGGD RLSNFMTWQT

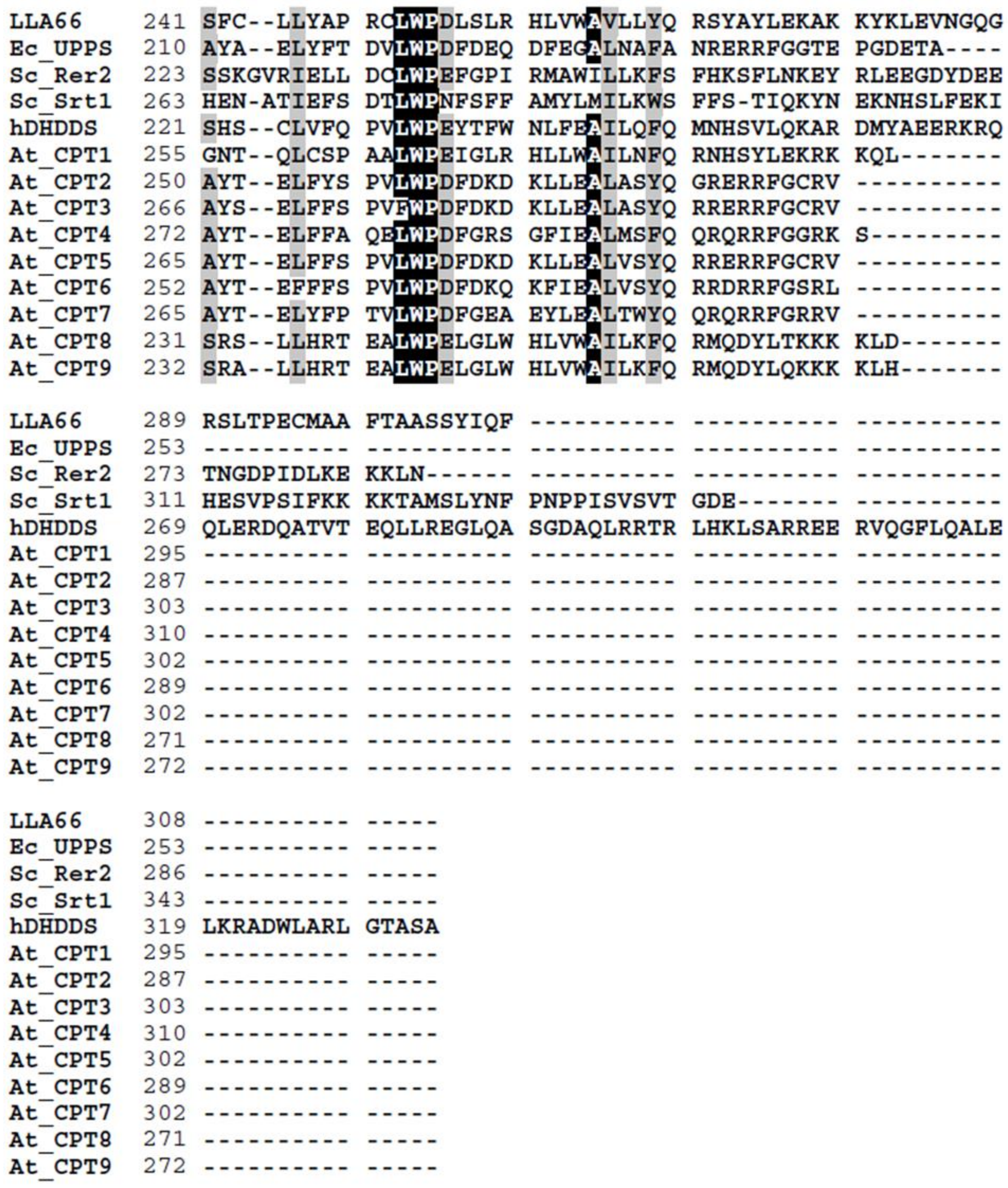

Figure S1. Sequence homology of LLA66 with other *cis*-prenyltransferases, including UPPS from *E. coli*, Rer2 and Srt1 from yeast, DHDDS from human, and 9 *cis*-prenyltransferases from *A. thaliana*.

(a)

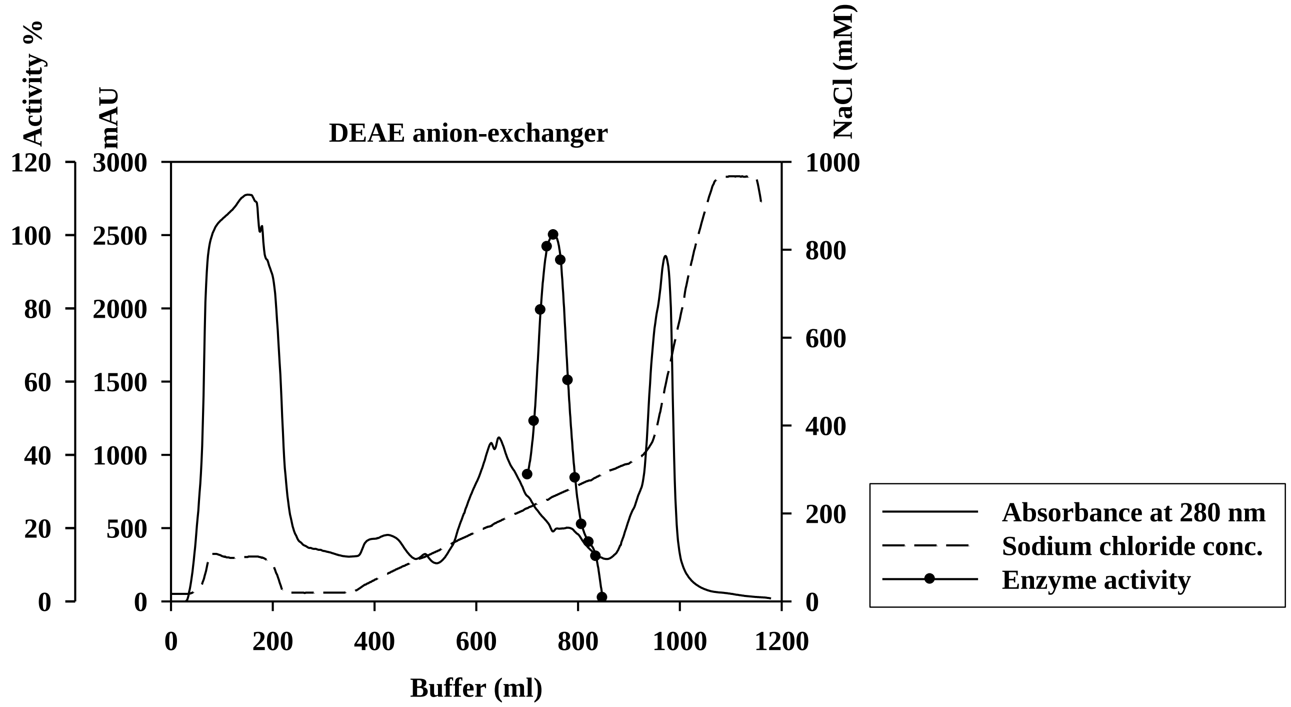
DEAE anion-exchanger
Activity %
mAU
NaCl (mM)
120 100 80 60 40 20 0
3000 2500 2000 1500 1000 500 0
1000 800 600 400 200 0
0 200 400 600 800 1000 1200
Buffer (ml)
Absorbance at 280 nm
Sodium chloride conc.
Enzyme activity

(b)

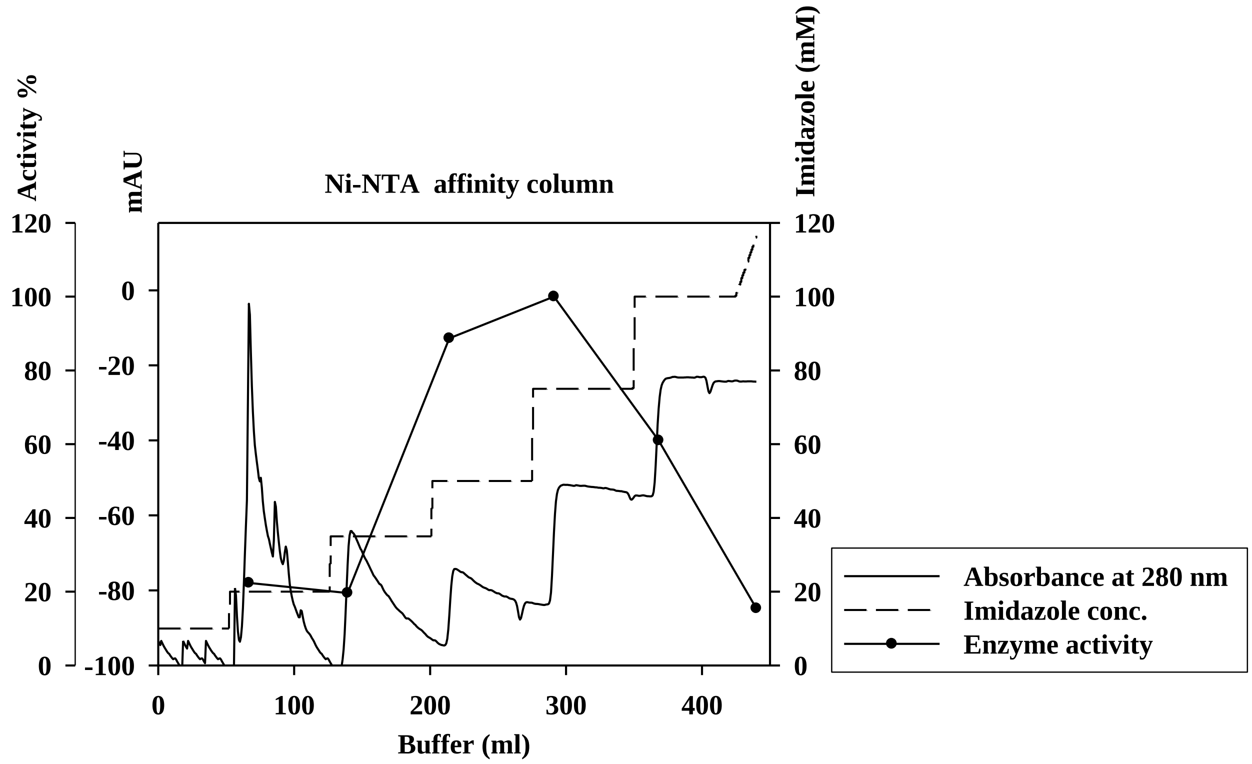
Ni-NTA affinity column
Activity %
mAU
Imidazole (mM)
120 100 80 60 40 20 0
0 -20 -40 -60 -80 -100
120 100 80 60 40 20 0
0 100 200 300 400
Buffer (ml)
Absorbance at 280 nm
Imidazole conc.
Enzyme activity

(c)

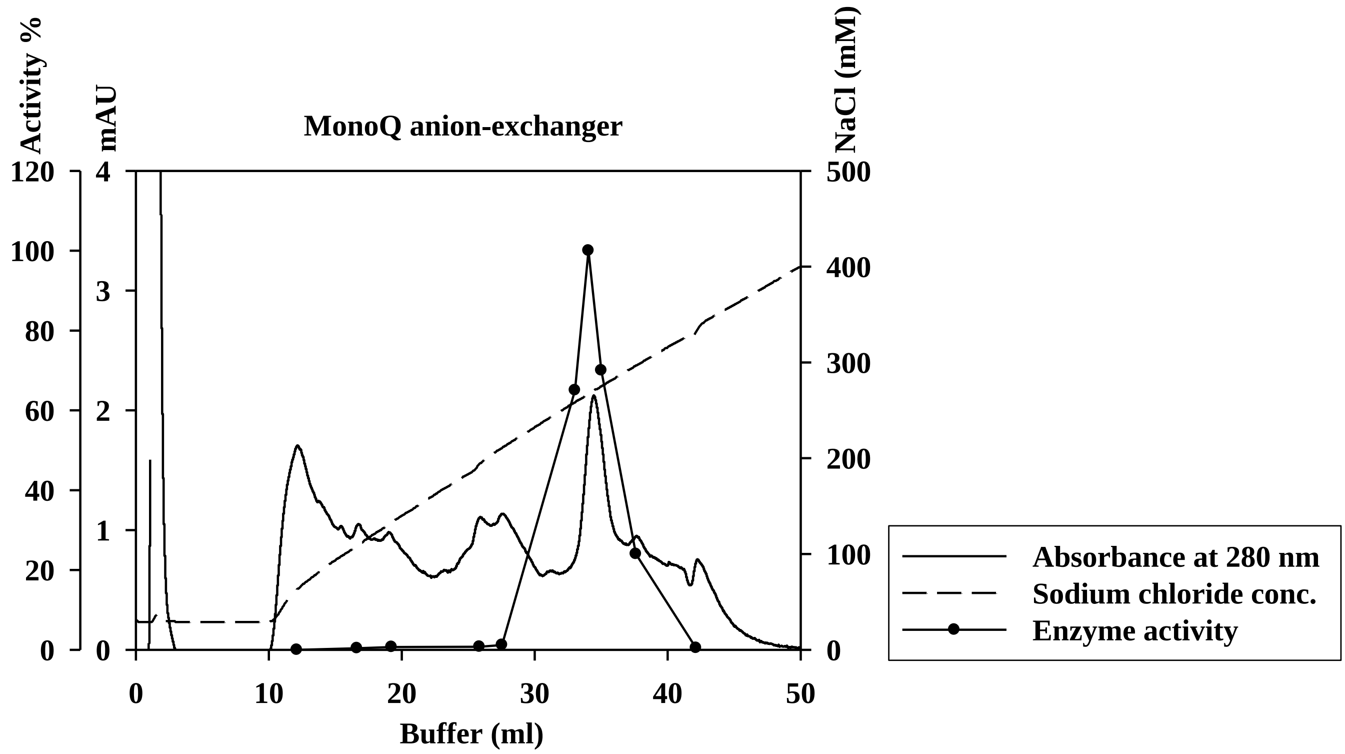
MonoQ anion-exchanger
Activity %
mAU
NaCl (mM)
120
100
80
60
40
20
0
4
3
2
1
0
500
400
300
200
100
0
0
10
20
30
40
50
Buffer (ml)
Absorbance at 280 nm
Sodium chloride conc.
Enzyme activity

(d)

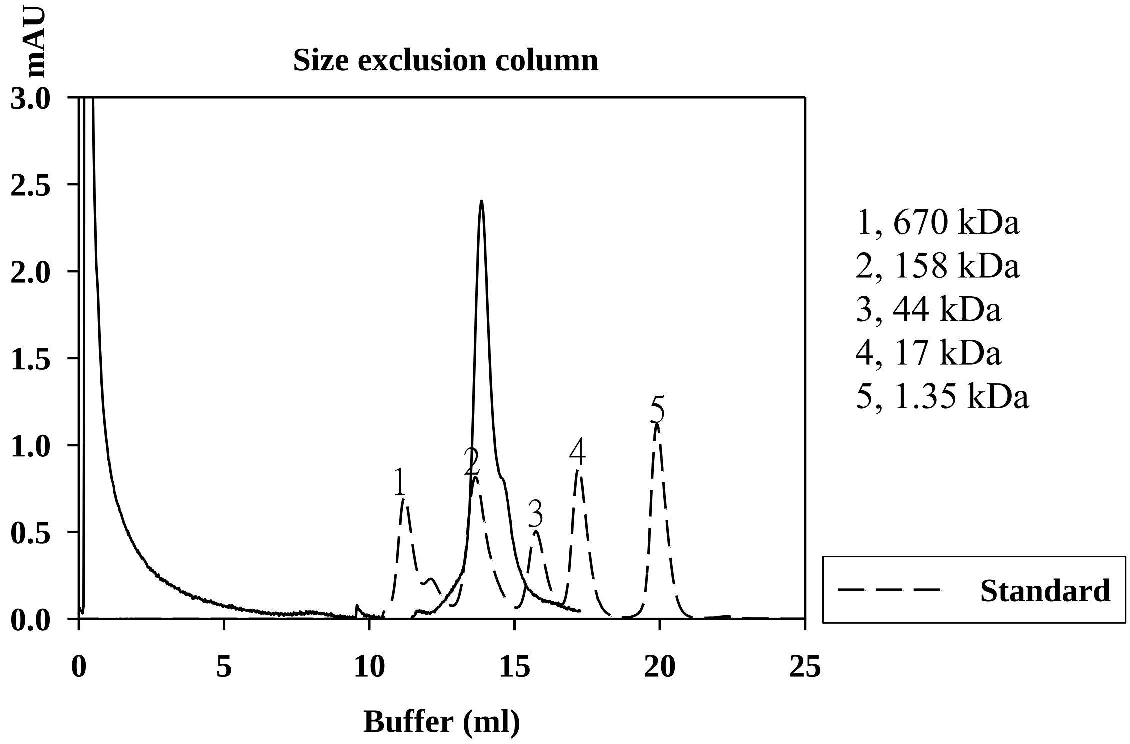
Size exclusion column
mAU
3.0
2.5
2.0
1.5
1.0
0.5
0.0
0
5
10
15
20
25
Buffer (ml)
1
2
3
4
5
1, 670 kDa
2, 158 kDa
3, 44 kDa
4, 17 kDa
5, 1.35 kDa
Standard

Figure S2. Purification of the *S. cerevisiae* enzyme complex that produced C15 farnesol from [^{14}C]IPP alone. *S. cerevisiae* cell lysate was loaded onto a DEAE anion exchanger and the IPP-utilizing activity appeared between 200 to 300 mM NaCl eluting fractions (a). These fractions were collected and loaded onto Ni-NTA column and the activity was found from the 100 mM imidazole elution (b). The active protein complex was further purified with anion exchanger Mono-Q (c), and size exclusion chromatography (d).

(a) **(b)**

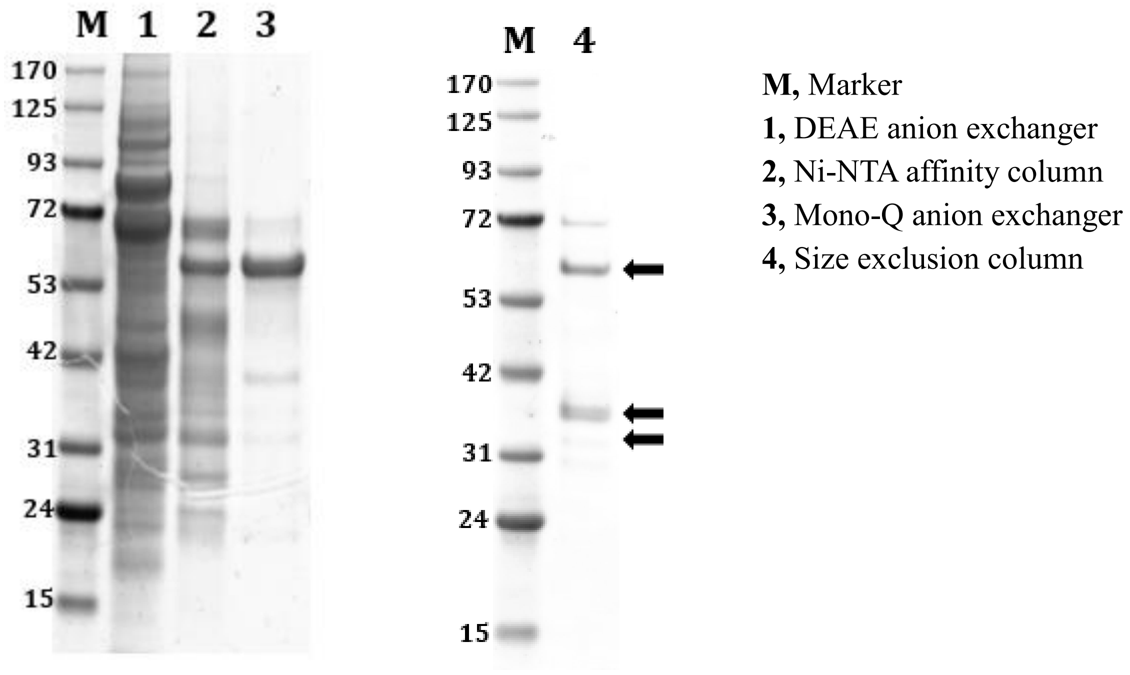

Figure S3. SDS-PAGE analysis of the *S. cerevisiae* enzyme complex from a series of purification steps. (a) and (b) show SDS-PAGE analysis of the proteins after the purification steps. Lane M, 1, 2, 3, and 4 indicate MW markers, after DEAE anion exchange column, Ni-NTA column, Mono-Q column, and size column, respectively. The arrows indicate the proteins taken for LC-MS/MS analysis.

(a)

1 MMTHTLPSEQ TRLVPGSDSS SRPKKRRISK RSKIIVSTVV CIGLLLVLVQ
51 LAFPSSFALR SASHKKK**NVI FFVTDGMGPA SLSMARSFNQ HVNDLPIDDI**
101 **LTLDEHFIGS SR**TR**SSDSLV TDSAAGATAF ACALKSYNGA IGVDPHHRPC**
151 **GTVLEAAKLA GYLTGLVVTT RITDATPASF SSHVDYRWQE DLIATHQLGE**
201 **YPLGRVVDLL MGGGRSHFYP QGEK**ASPYGH HGARK**DGRDL IDEAQSNGWQ**
251 **YVGDRKNFDS LLK**SHGENVT LPFLGLFADN DIPFEIDRDE K**EYPSLK**EQV
301 K**VALGALEK**A SNEDK**DSNGF FLMVEGSRID HAGHQNDPAS QVR**EVLAFDE
351 AFQYVLEFAE NSDTETVLVS TSDHETGGLV TSRQVTASYP QYVWYPQVLA
401 NATHSGEFLK R**KLVDFVHEH K**GASSK**IENF IKHEILEKDL GIYDYTDSDL**
451 **ETLIHLDDNA NAIQDKLNDM VSFRAQIGWT THGHSAVDVN IYAYANKK**AT
501 WSYVLNNLQG NHENTEVGQF LENFLELNLN EVTDLIRDTK HTSDFDATEI
551 ASEVQHYDEY YHELTN

(b)

1 MASEKEIRRE R**FLNVFPKLV EELNASLLAY GMPK**EACDWY AHSLNYNTPG
51 GK**LNRGLSVV DTYAILSNKT VEQLGQEEYE K**VAILGWCIE LLQAYFLVAD
101 DMMDKSITRR GQPCWYKVPE VGEIAINDAF MLEAAIYKLL KSHFRNEKYY
151 IDITELFHEV TFQTELGQLM DLITAPEDKV DLSKFSLKK**H SFIVTFK**TAY
201 YSFYLPVALA MYVAGITDEK DLKQARDVLI PLGEYFQIQD DYLDCFGTPE
251 QIGK**IGTDIQ DNK**CSWVINK **ALELASAEQR** K**TLDENYGK**K DSVAEAKCKK
301 IFNDLK**IEQL YHEYEESIAK** DLKAK**ISQVD ESRGFKADVL TAFLNK**VYKR
351 SK

(c)

1 **MTADNNSMPH GAVSSYAKLV QNQTPEDILE EFPEIIPLQQ R**PNTRSSETS
51 NDESGETCFS GHDEEQIKLM NENCIVLDWD DNAIGAGTKK **VCHLMENIEK**
101 GLLHRAFSVF IFNEQGELLL QQRATEKITF PDLWTNTCCS HPLCIDDELG
151 LKGKLDDKIK GAITAAVR**KL DHELGIPEDE TK**TRGK**FHFL NRIHYMAPSN**
201 **EPWGEHEIDY ILFYK**INAK**E NLTVNPNVNE VR**DFKWVSPN DLK**TMFADPS**
251 **YK**FTPWFKII CENYLFNWWE QLDDLSEVEN DRQIHRML

Figure S4. Identification of the proteins from LC-MS/MS analysis. After the protease digestions, the peptides detected (shown in red) represented alkaline phosphatase (a), FPPS (b), and IPP:DMAPP isomerase (c).

Table S1. Proteins identified by LC-MS/MS analysis from the protease digested SDS-PAGE bands

Bands on Gel	Protein hits	MW	Score/peptides/coverage
~60 kDa band	alkaline phosphatase	62965	3031/122/54%
~40 kDa band	Farnesyl pyrophosphate synthase	40458	1753/56/34%
~30 kDa band	IPP:DMAPP isomerase	33330	579/39/41%
